# Complications of preventive loop ileostomy versus colostomy: a meta-analysis, trial sequential analysis, and systematic review

**DOI:** 10.1186/s12893-023-02129-w

**Published:** 2023-08-12

**Authors:** Zheng Ge, Xiang Zhao, Zitian Liu, Guangwei Yang, Qunzheng Wu, Xiaoyang Wang, Xiang Zhang, Zhiqiang Cheng, Kexin Wang

**Affiliations:** 1 Cheeloo College of Medicine, Shandong University, Jinan, Shandong China; 2 Department of General Surgery, Qilu Hospital of Shandong University, Jinan, Shandong China

**Keywords:** Preventive loop ileostomy, Preventive loop colostomy, Prolapse, Complication

## Abstract

**Background:**

Preventive colostomy is required for colorectal surgery, and the incidence of complications associated with ileostomy and colostomy remains controversial. This study aimed to compare the incidence of postoperative complications between ileostomy and colostomy procedures.

**Methods:**

Data analysis was conducted on 30 studies, and meta-analysis and trial sequential analysis (TSA) were performed on five studies. The basic indicators, such as stoma prolapse, leak, wound infection, ileus, and a series of other indicators, were compared.

**Results:**

No statistically significant differences were observed with complications other than stoma prolapse. Meta-analysis and TSA showed that the incidence of ileostomy prolapse was lower than that of colostomy prolapse, and the difference was statistically significant. Apart from the four complications listed above, the general data analysis showed differences in incidence between the two groups. The incidence of skin irritation, parastomal hernia, dehydration, pneumonia, and urinary tract infections was higher with ileostomy than with colostomy. In contrast, the incidence of parastomal fistula, stenosis, hemorrhage, and enterocutaneous fistula was higher with colostomy than with ileostomy.

**Conclusions:**

There were differences in the incidence of ileostomy and colostomy complications in the selected studies, with a low incidence of ileostomy prolapse.

**PROSPERO registration number:**

CRD42022303133.

**Supplementary Information:**

The online version contains supplementary material available at 10.1186/s12893-023-02129-w.

## Introduction

In recent years, the incidence of rectal cancer has increased annually [1]. Consequently, anus-preserving surgery is frequently performed in patients with low rectal cancer, especially those with advanced ultralow rectal cancer. However, postoperative healing is worse, and the frequency of anastomotic leakage greatly increases with a lower anastomotic stoma.

This problem can be resolved with the use of preventive stomas [2]. Although evidence suggests that preventive stomas do not reduce the incidence of leakage, they reduce the severity of pelvic infections after anastomotic leakage occurs and consequently reduce the rate of secondary surgery. Moreover, preventive stomas do not increase the surgery difficulty or the patient’s length of hospital stay.

While a well-functioning stoma can significantly improve a patient’s quality of life, it is a nonphysiological condition that can inevitably lead to many complications, such as stoma prolapse, retraction, bleeding, necrosis, and hernia. The incidence of these complications ranges approximately from 21 to 70% [3]. There are two types of preventive stomas: ileostomy and colostomy. Each has advantages and disadvantages, such as complication rates, patient acceptance, and quality of life.

This study assessed a total of 30 papers, including five randomized control trials (RCTs), to further investigate the incidence of stoma complications. Thereafter, this study used a meta-analysis and trial sequential analysis (TSA) to study the complications of ileostomy and colostomy, as well as a systematic review of the 30 publications to study the incidence of these complications.

## Methods

### Literature search and screening

A systematic review of the literature published through October 2022 was performed by searching for abstracts in conference papers and the MEDLINE, EMBASE, Cochrane Library, and Clinicaltrials.gov databases. Medical subject headings and keywords were used for the search, and this study included RCTs and excluded non-comparative studies, reviews, and descriptive research.

Retrieval strategy (PubMed): Search: ((((ileostomy) OR (loop ileostomy)) OR (colostomy)) OR (loop colostomy)) AND (complication) Filters: Randomized controlled trial.

The search results were reviewed manually, and disagreements were discussed and resolved by all the authors.

The inclusion criteria were: (1) studies that compared “ileostomy” versus “colostomy” or only analyzed data on “ileostomy” or “colostomy”; (2) studies that reported the perioperative outcomes and postoperative complications of stoma surgery; and (3) RCTs.

The exclusion criteria were: (1) studies that did not distinguish between “ileostomy” and “colostomy,” or the combinations with data that were difficult to extract; and (2) studies without complete data, evaluation criteria for complications, or exact complication rates.

### Data extraction and quality assessment

Data from the published articles, including baseline characteristics and incidence of complications, were collected using Microsoft Excel. Baseline characteristics included the authors’ names, publication time, number of patients, age, sex, overall mortality, tumor size, intraoperative blood loss, operative time for stoma formation and closure, time to ileostomy closure, and length of hospital stay. The complication rate was the main study index, and complications included prolapse, retraction, skin irritation, parastomal hernia, parastomal fistula, incisional hernia, high output, leak, stenosis, wound sepsis, bleeding, wound infection, enterocutaneous fistula, intestinal obstruction/ileus, pneumonia, and urinary tract infections. Two reviewers independently extracted the data and consulted each other in case of disagreement. The quality assessment of the selected studies was based on *the Cochrane Collaboration’s tool for assessing the risk of bias in randomized trials*.

### Statistical analysis

The statistical data were analyzed using reviewer manager 5.4 (Cochrane Collaboration, Oxford, UK) and SPSS version 18.0 (SPSS, Chicago, IL, United States). A binary variable was used to compare the incidence of complications between the ileostomy and colostomy groups. Heterogeneity was assessed using the inconsistency index (I^2^), with values of 0–25%, 25–75%, and 75–100% representing low, moderate, and substantial heterogeneity, respectively. It is generally accepted that if I^2^ is relatively low while assuming a fixed value for the theoretical effect size, a fixed-effects model is used; otherwise, if I^2^ indicates significant heterogeneity, a random-effects model is used. The Mantel-Haenszel method was used to analyze the data and set the odds ratio (OR) as the effect size with a 95% confidence interval (CI). Forest and funnel plots were also obtained. “Favorable ileostomy” was considered when 95% CI of OR was < 1, and “favorable colostomy” was considered on the contrary.

### Trial sequential analysis

TSA was used in this study for the sensitivity analysis of complication rates, correcting random errors, and quantifying the information size (IS). A binary variable was used for the analysis, and a fixed-effects model was applied. TSA was performed using the TSA software (0.9.5.10 Beta, Copenhagen Trial Unit, Denmark).

The original TSA data were the same as those used in the meta-analysis. In addition, conventional test boundaries and alpha-spending boundaries were added to the data analysis for the significance test. The conventional option allowed the addition of a boundary for the Z-curve, which corresponded to a single significance test with a maximum type-I error risk, alpha (α). The α-spending option allowed the addition of adjusted significance boundaries for the Z-curve using the α-spending method. The required IS was calculated for each variable based on a 5%-value for α and 20% for beta (β) (equal to 80% power) using the O’Brien–Fleming function.

Using TSA prevented the occurrence of false-positive results due to the lack of sample size and led to a more accurate conclusion. For example, if the cumulative Z-curve exceeded the IS or intersected with the O’Brien-Fleming boundaries, the difference between “ileostomy” and “colostomy” showed firm or weak evidence.

## Results

### Literature search and study characteristics

**Fig. 1:**
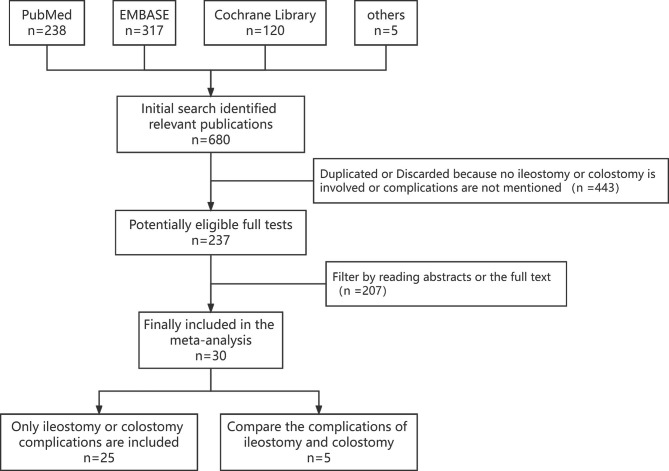
PRISMA literature search flow chart

A total of 680 RCTs were identified from the database following the retrieval strategy. Of these, 650 were excluded based on the inclusion and exclusion criteria. Thus, this study included 30 RCTs, and five were compared for the complication rates of ileostomy and colostomy procedures (Fig. 1). Among these five studies, three were from the United Kingdom, while two were from the Netherlands and China.

There were no differences in study characteristics observed among these five studies (Table 1).

**Table 1:** Study characteristics of five studies comparing the complication rates of ileostomy and colostomy

Reference	Design	No. of patients	Sample size		Mean age (years)		Male N (%)		Days to stoma closure Median (range)	
			Ileostomy	Colostomy	Ileostomy	Colostomy	Ileostomy	Colostomy	Ileostomy	Colostomy
Law 2002^4^	RCT	80	42	38	65.2	67.8	26 (61.90)	23 (60.53)	183	180
Edwards 2001^5^	RCT	70	34	36	63	68	27 (79.41)	22 (61.11)	62 (17–120)	73 (28 ± 141)
Gooszen 1998^6^	RCT	76	37	39	63.2	64.7	14 (37.84)	13 (33.33)	-	-
Khoury 1987^7^	RCT	61	32	29	65	65	23 (47.83)	13 (50.00)	15.0 (10–64)	19.0 (9–138)
Williams 1986^8^	RCT	47	23	24	71	66.5	11 (71.88)	12 (44.83)	-	-

### Quality assessment results

**Fig. 2:**
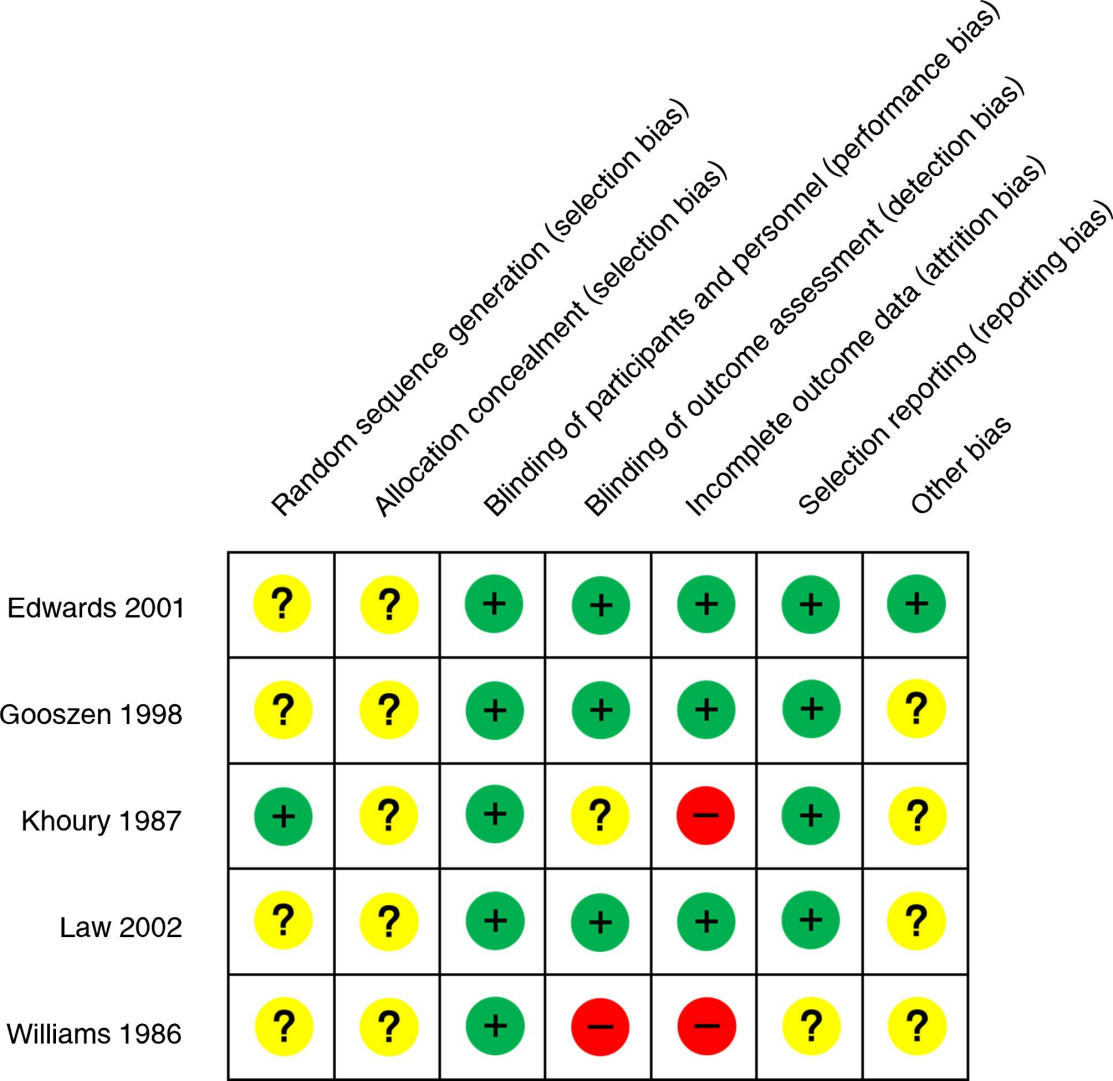
Risk of bias summary

The quality assessment results are shown in Fig. 2. The interventions in this study were ileostomy and colostomy, which cannot be performed using the blinding method. Therefore, these interventions were regarded as low-risk interventions. However, the early publication of some literature resulted in articles with unclear experimental methods, incomplete statistical results, and partially missing follow-up records. These inconsistencies led to an increased risk of attrition and detection biases.

### Complications

#### Prolapse

**Fig. 3:**
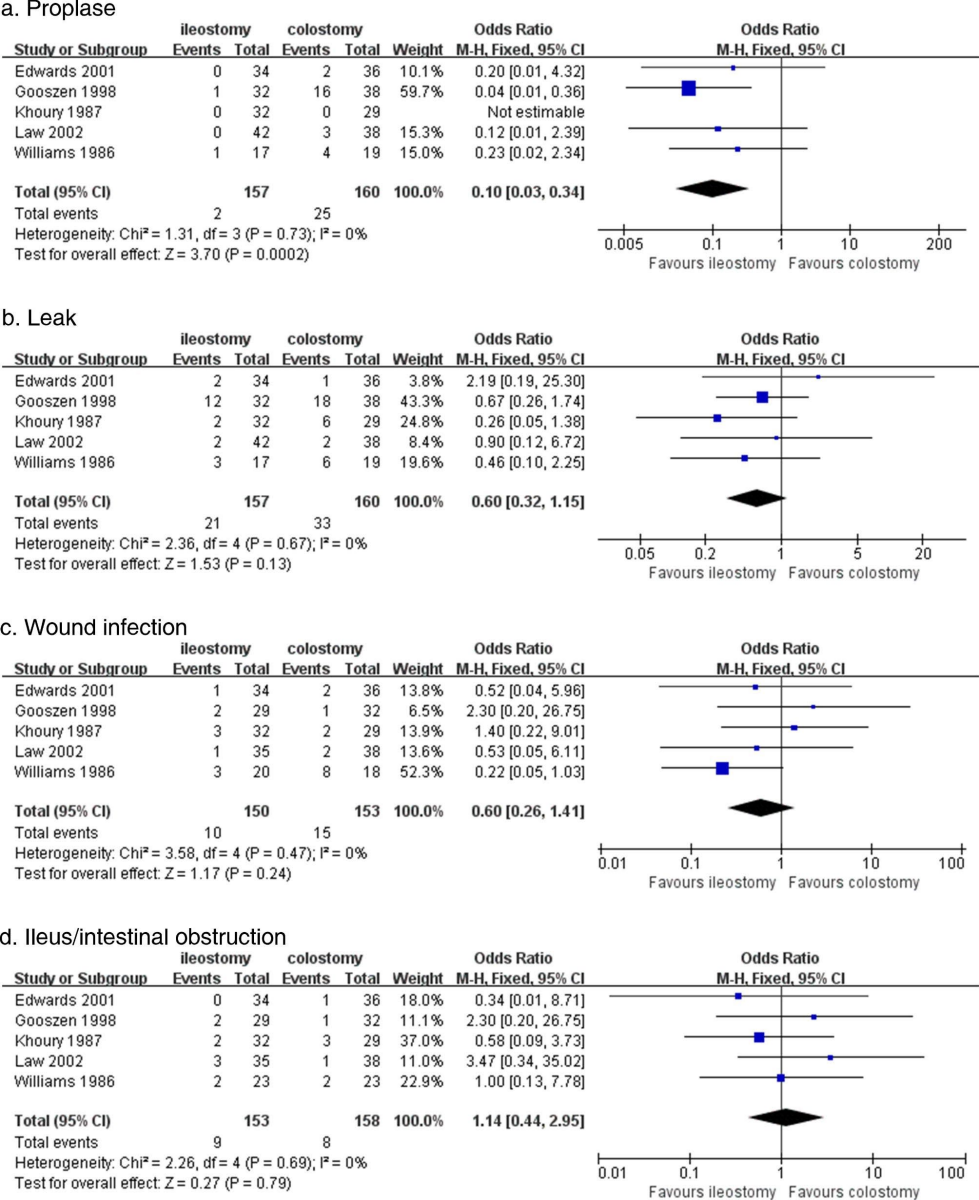
Forest plots of four complications

All five studies, including 317 patients (157 with ileostomy and 160 with colostomy), reported the complication rate of prolapse; I^2^ = 0% meant there was no heterogeneity, and a fixed-effects model was used. The total prolapse rate was 8.51%, with 1.27% (2/157) for ileostomy and 15.63% (25/160) for colostomy. There was a significant difference in the prolapse rate between the ileostomy and colostomy groups (OR (95% CI): 0.10 (0.03–0.34); p = 0.0002), meaning that the prolapse rate in the ileostomy group was lower than that in the colostomy group (Fig. 3a).

#### Leak

All five studies, including 317 patients (157 with ileostomy and 160 with colostomy), reported the complication rate of leaks; I^2^ = 0% meant there was no heterogeneity, and a fixed-effects model was used. The total leakage rate was 17.03%, with 13.38% (21/157) for ileostomy and 20.63% (33/160) for colostomy. There was no significant difference in the leak rate between the ileostomy and colostomy groups [OR (95% CI): 0.60 (0.32–1.15); p = 0.13] (Fig. 3b).

#### Wound infection

All five studies, including 303 patients (150 with ileostomy and 153 with colostomy), reported the complication rate of wound infection; I^2^ = 0% meant there was no heterogeneity, and a fixed-effects model was used. The total wound infection rate was 8.25%, with 6.67% (10/150) for ileostomy and 9.80% (15/153) for colostomy. There was no significant difference in the wound infection rate between the ileostomy and colostomy groups [OR (95% CI): 0.60 (0.26–1.41); p = 0.24] (Fig. 3c).

#### Ileus/intestinal obstruction

All five studies, including 311 patients (153 with ileostomy and 158 with colostomy), reported the complication rate of ileus; I^2^ = 0% meant there was no heterogeneity, and a fixed-effects model was used. The total ileus rate was 5.47%, with 5.88% (9/153) for ileostomy and 5.06% (8/158) for colostomy. There was no significant difference in ileus rate between the ileostomy and colostomy groups [OR (95% CI): 1.14 (0.44–2.95); p = 0.79] (Fig. 3d).

#### Publication bias

**Fig. 4:**
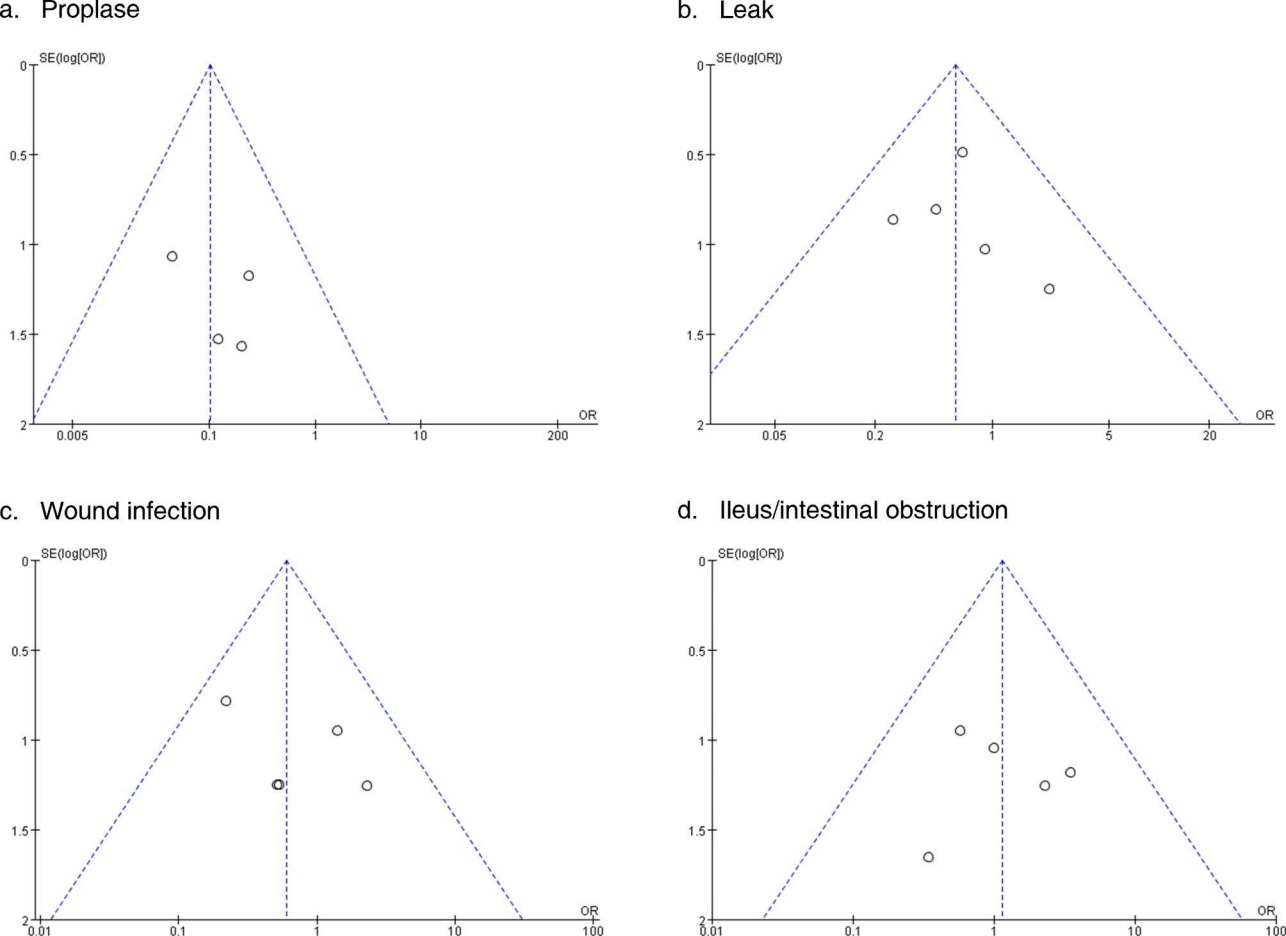
Funnel plot of four complications

Visual inspection of the funnel plot (Fig. 4) showed no evidence of publication bias for any of the designated variables in any of the included studies (p > 0.05).

#### Trial sequential analysis

**Fig. 5:**
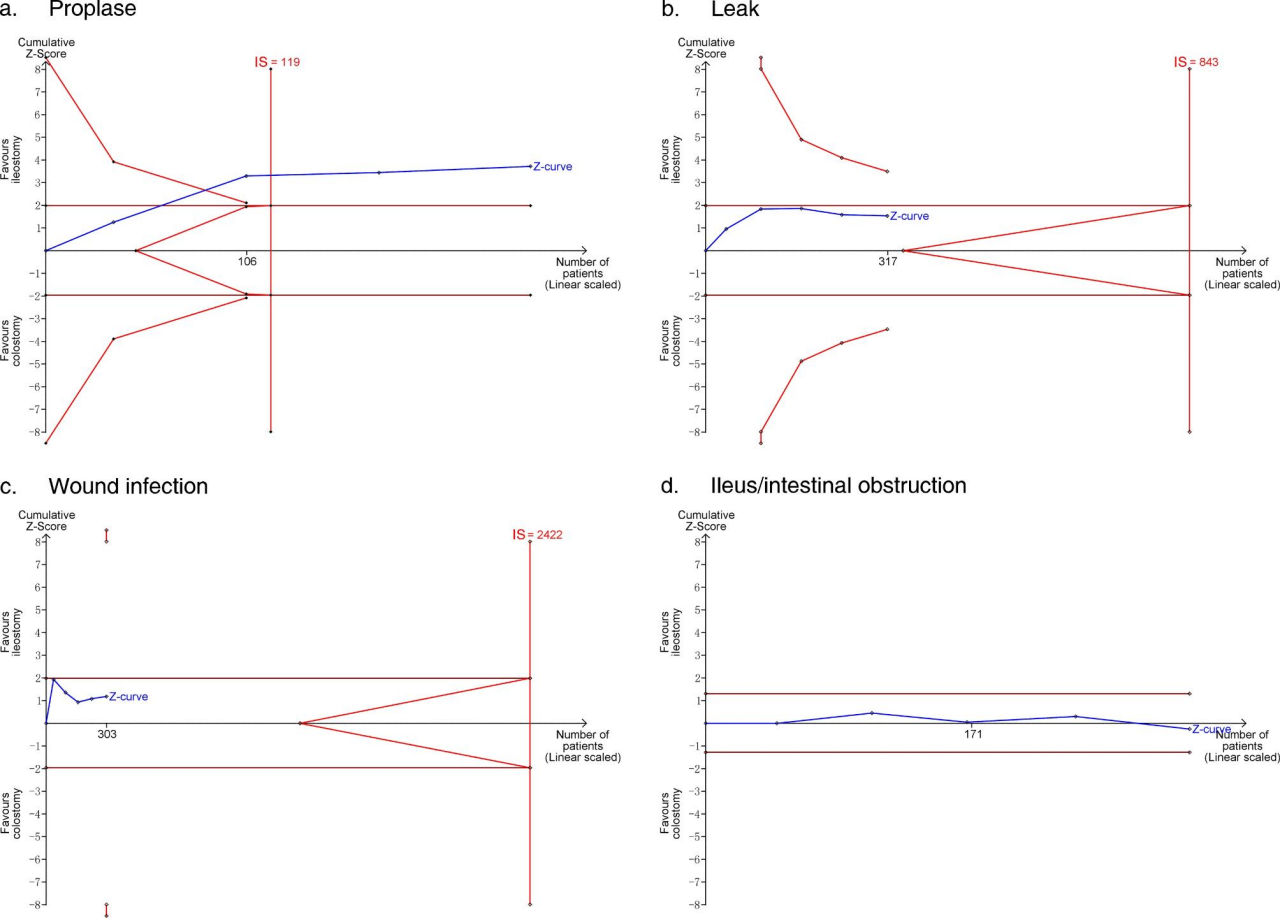
Plot of TSA analysis results for four complications

TSA analysis was performed for prolapse, leakage, wound infection, and ileus (Fig. 5). Among these four complications, the Z-curves of a leak, wound infection, and ileus did not exceed the IS or intersect with the O’Brien-Fleming boundaries, meaning the difference between “ileostomy” and “colostomy” did not show firm evidence. Only the Z-curve of prolapse exceeded the IS and intersected with the O’Brien-Fleming boundaries, which showed firm evidence of this difference.

#### Expanded data analysis

In addition to the meta-analysis and TSA of these indexes, a comparative analysis of the 30 RCTs was also performed [4–28]. Twenty-five of these RCTs only studied loop colostomy or ileostomy. In addition, only the chi-square test was performed for complication rate because of the limitations of sample size and differences between surgeons and hospitals. The results of this analysis are shown in Table 2.

**Table 2:** Comparative analysis of the complication rates in randomized control trials using the chi-squared test

	Ileostomy	Colostomy	p-value	Method
prolapse	0.942(5/535)	7.837(50/638)	< 0.001	Chi-Square Test
retraction	7.324(35/478)	6.816(55/807)	0.749	Chi-Square Test
skin irritation	56.888(272/478)	31.439(166/528)	< 0.001	Chi-Square Test
parastomal hernia	4.5(12/267)	3.644(18/494)	0.581	Chi-Square Test
parastomal fistula	0.791(5/633)	3.846(4/104)	0.036	Fix Chi-Square test
incisional hernia	5.086(6/118)	5.914(11/186)	0.772	Chi-Square Test
high output	7.27(57/784)	0.895(1/112)	0.014	Chi-Square Test
leak	4.4(70/1591)	8.123(74/911)	< 0.001	Chi-Square Test
stenosis	3.429(24/700)	4.627(26/562)	0.298	Chi-Square Test
wound sepsis	4.572(30/656)	4.762(9/189)	0.917	Chi-Square Test
bleeding	1.344(11/819)	2.473(7/283)	0.204	Chi-Square Test
wound infection	9.194(122/1327)	10.505(127/1209)	0.315	Chi-Square Test
enterocutaneous fistula	2.499(11/440)	6.202(16/258)	0.019	Chi-Square Test
intestinal obstruction / ileus	11.06(212/1917)	2.964(15/506)	< 0.001	Chi-Square Test
pneumonia	3.941(27/685)	2.044(14/685)	0.045	Chi-Square Test
urinary tract infection	3.07(19/619)	2.318(7/302)	0.529	Chi-Square Test

## Discussion

To the best of our knowledge, although several similar meta-analyses have compared ileostomy and colostomy complications [29, 30], this is the first meta-analysis that included TSA analysis. This study shows strong evidence of a lower incidence of prolapse for ileostomy than for colostomy [3, 31]. However, for the other three complications, including wound infection, fistula, and intestinal obstruction, weak evidence showed no difference between ileostomy and colostomy. In addition, the difference in the incidence of stoma complications between the two groups was also compared using 30 RCT studies on stoma complications.

With the high incidence of colorectal cancer, the utilization rate of ostomy is also increasing annually [1, 32]. Approximately 725 to 1 million people in the United States have undergone ostomies. In China, the number of people undergoing permanent ostomies has exceeded 1 million, and the number is rapidly increasing by 100,000 annually. In a 1998 study, these two types of stoma were used equally: 36.1% for colostomy and 32.2% for ileostomy (and 31.7% for urostomy). However, ileostomy is currently more frequently used than colostomy. Owing to the different positions of the stoma tube in the digestive tract, there are significant differences in the characteristics and flow rates of the diversion. Therefore, the incidence of complications between the two groups should have also been different.

Stoma prolapse refers to the protrusion of the intestinal stoma loop through the stoma, which is more common in loop stomas. Symptoms such as edema, bleeding, ulcers, and incarceration can be observed in the protruding bowel [31]. Excessive abdominal wall defects have been identified as potential causes of stoma retraction, prolapse, and early hernia formation. A meta-analysis study has demonstrated that utilizing preventive mesh reinforcement at the ostomy site can be a safe and effective approach, with a low incidence of stoma site incisional hernia (SSIH) [33]. Age, obesity, and increased intra-abdominal pressure are risk factors for prolapse [34, 35]. The data indicated that the incidence of prolapse was lower for the ileostomy group than for the colostomy group, and the TSA analysis indicated a significant difference. There is currently no high-level evidence in the literature to explain this phenomenon. However, the authors speculate that there are several reasons for this finding. First, the walls of the small intestine and colon are the same and are composed of mucosal, submucosal, muscular, and serosal layers. However, the wall thickness of the normally filled small intestine is < 3 mm, and the colon wall is slightly thicker than that of the small intestine; therefore, it may be more prone to prolapse. Second, the stoma opening size may also be an influencing factor of stoma prolapse. The incidence of prolapse differs between colostomy and ileostomy due to the thickness of the colonic lumen compared to the small intestine and the surgeon’s habits. In addition, stool in the colon is a semi-solid material that moves faster than the peristaltic waves in the ileum. Geng, Nasier [36] suggested that this phenomenon makes poststomal colonic prolapse more likely to occur than ileal prolapse. In the case of stoma prolapse, nonsurgical or surgical treatment should be performed according to the degree of retraction, as appropriate. Nonsurgical treatment can be used to subdue edema, such as bed rest, wet compression with hypertonic solution, and manual reduction. Emergency surgery should be performed in patients with stoma prolapse who have volvulus, obstruction, or ischemia. Surgical treatment should be considered in patients who cannot undergo repair. After removing the prolapsed intestinal segment, the stoma should be reconstructed in a suitable position.

In addition to the above comparison items, other complication rates were compared. However, because these complications were not fully addressed in these five RCTs, the scope of the included literature was expanded to include all RCTs that investigated the complications of ileostomy and colostomy. A total of 25 RCTs that examined the incidence of other interventions (e.g., early temporary ileostomy versus standard closure in patients with rectal cancer [26]) limited to ileostomy or colostomy were added. Due to differences in surgeons, medical conditions, diagnosis, and treatment processes, a meta-analysis was not performed, and only the chi-square test was performed. A difference in incidence rates > 20% was considered the difference, and the higher incidence rates were marked. The results are shown in the Table 2. According to the conclusions in the Table 2, the incidence of colostomy prolapse was significantly higher than that of ileostomy, which is consistent with the conclusions of the meta-analysis. Although there was no difference in the incidence of wound infection and leak in the meta-analysis, the incidence of colostomy was higher than that of ileostomy, and the incidence of colostomy was also higher in the Table 2. However, the difference in wound infection was not significant. In the meta-analysis, the incidence of ileus was similar between the two groups. However, the incidence of ileus in the ileostomy group was higher than that of the colostomy group, as shown in the Table 2. Hence, more evidence is still required to prove this conclusion.

Except for the four complications stated above, the general data analysis showed differences in the incidence of complications between the two groups. The incidence of skin irritation, parastomal hernia, dehydration, pneumonia, and urinary tract infections was higher for ileostomy. In contrast, the incidence of parastomal fistula, stenosis, hemorrhage, and enterocutaneous fistula was higher for colostomy. For skin irritation and dehydration, probably because the colonic and ileal contents are different, the colon has a strong absorptive capacity and can absorb more than 5 L of fluid and electrolytes daily. For patients with ileostomy, the output increases in the early stage and normalizes in the later stage; this process lasts for 1–8 weeks [37]. However, a sustained ileostomy output of > 1500 ml may lead to dehydration, electrolyte imbalance, and acute kidney injury. Dehydration is the most common cause of readmission [38]. The pH of the intestinal fluid is relatively alkaline and contains digestive enzymes. When directly exposed to the skin, it causes skin irritation symptoms, such as redness, ulceration, itching, and pain, resulting in a higher incidence of skin irritation in ileostomy than in colostomy.

Moreover, based on actual clinical experience, it has been observed that patients with transverse colostomy often express concerns about the social inconvenience caused by the odor of stoma excreta. Conversely, individuals with ileostomy commonly face challenges related to stoma and skin care due to the thin, large, and irritating nature of their excreta. In addition, the specific type of ostomy surgery can also have a impact. A study has demonstrated that creating skin bridge loop stoma leads to improved early stoma management, enhanced adhesion of stoma appliances, and ultimately, a better quality of life for the patient [39, 40]. However, improvements have been made in addressing these issues through advancements in stoma products, enhanced stoma care practices, and the guidance provided by professional stoma doctors. In cases where a permanent stoma is required, colostomy is generally preferred due to its lower excreta output, which helps reduce the risk of dehydration and electrolyte imbalances.

The advantages of this study are that it updates the previous meta-analyses, consolidates the conclusions using TSA analysis, adopts stricter quality control, and includes more RCT studies to obtain more abundant conclusions. However, this study had some limitations. First, the study included only five comparative RCTs, including 317 participants (157 with ileostomy and 160 with colostomy). The small number of RCTs, small number of participants, and low incidence of complications may be one of the reasons for only partially positive conclusions. Twenty-five high-quality RCTs were included for comparison, with a total of 3679 participants (1977 with ileostomy and 1702 with colostomy) to solve this problem. The other aspects of the conclusions were positive, but owing to differences in operators, medical conditions, and diagnosis and treatment processes, the evidence level of this aspect was low. Second, five RCTs included in this study were conducted at a relatively early age, and the description of experimental and bias control methods was not precise; hence, the control of data bias could not be guaranteed. In recent years, few comparative RCT studies of ileostomy and colostomy have been published, making it difficult for some conclusions that have become an expert consensus to be confirmed by a higher level of evidence.

## Conclusion

In this study, we compared the complication rates of ileostomy and colostomy through parameter testing. Our findings revealed distinct differences in the incidence of various complications between patients with ileostomy and colostomy. Additionally, the meta-analysis indicated that, apart from a lower occurrence of colostomy prolapse in the ileostomy group compared to the colostomy group, there were no significant differences in complication rates between the two groups. More high-quality RCTs are required to conclude with more significant differences in the incidence of complications.

### Electronic supplementary material

Below is the link to the electronic supplementary material.

Additional File 1: PRISMA 2020 Checklist

## Data Availability

The datasets generated and analysed during the current study are available in the MEDLINE, EMBASE, Cochrane Library, and Clinicaltrials.gov databases.
